# Prevalence of Psychiatric and Addictive Disorders in Patients with Psoriasis: A Cross-Sectional Study

**DOI:** 10.3390/diagnostics15101231

**Published:** 2025-05-14

**Authors:** Daciana Elena Brănișteanu, Roxana Paraschiva Ciobanu, Daniel Constantin Branisteanu, Cristina Colac-Boțoc, Antonia-Elena Huțanu, Cătălina-Anca Munteanu, Rares Stamate, George Brănișteanu, Catalina Ioana Onu-Branisteanu, Mihaela Paula Toader, Elena Porumb-Andrese

**Affiliations:** 1 Discipline of Dermatology, Grigore T. Popa University of Medicine and Pharmacy, 16 Universitatii Str., 700115 Iasi, Romaniaelena.andrese1@umfiasi.ro (E.P.-A.); 2 Dermatology Clinic, Railways University Hospital, 1 Garabet Ibraileanu Str., 700115 Iasi, Romania; 3 Discipline of Ophthalmology, Grigore T. Popa University of Medicine and Pharmacy, 16 Universitatii Str., 700115 Iasi, Romania; 4 Ophthalmology Clinic, Railways University Hospital, 1 Garabet Ibraileanu Str., 700115 Iasi, Romania; 5 Institute of Psychiatry “Socola”, Bucium Street, No 36, 700282 Iasi, Romania; 6 Orthopedy Clinic, Recovery Hospital, 14 Pantelimon Halipa Str., 700661 Iasi, Romania; 7 Institute for Cardiovascular Diseases C.C. Iliescu, 258 Fundeni Str., 022328 Bucharest, Romania; 8 Discipline of Oral Medicine, Oral Dermatology, Grigore T. Popa University of Medicine and Pharmacy, 16 Universitatii Str., 700115 Iasi, Romania

**Keywords:** psoriasis, depression, anxiety, substance use disorders, alcohol use disorder, tobacco use disorder, binge eating, inflammation, psychodermatology

## Abstract

**Background/Objectives:** Psoriasis is a chronic inflammatory skin disease increasingly linked to psychiatric and behavioral comorbidities, including depression, anxiety, and substance use disorders. Shared inflammatory pathways, including elevated IL-6, TNF-α, and IL-17, may link psoriasis with psychiatric disorders such as depression and anxiety. The bidirectional interaction between systemic inflammation and mental health may exacerbate the disease burden and affect treatment outcomes. The objective of this study was to determine the prevalence of psychiatric and behavioral comorbidities in patients with psoriasis and to explore potential demographic and clinical correlations. Assessing these correlations contributes to a better understanding of the mental health status of psoriasis patients, potentially influencing both therapeutic efficacy and quality of life. **Methods:** We conducted a cross-sectional observational study on 316 patients with clinically and histopathologically confirmed psoriasis, evaluated between January 2021 and March 2025 at the Clinical Railway Hospital in Iași, Romania. Psychiatric and behavioral comorbidities were assessed through clinical interviews, medical record reviews, and standardized tools including AUDIT-C, Fagerström Test for Nicotine Dependence, and the Binge Eating Scale. Psoriasis severity was evaluated using the Psoriasis Area and Severity Index (PASI). **Results:** Of 316 participants, 88 (27.8%) had psychiatric/behavioral comorbidities. The most frequent conditions were tobacco use disorder (11.1% overall; 39.8% among comorbid patients), alcohol use disorder (9.2%; 32.9%), binge eating (7.9%; 28.4%), anxiety (6.3%; 22.7%), and depression (4.1%; 14.8%). Additional diagnoses included personality disorders, dementia, PTSD, and sleep disorders. **Conclusions:** Psychiatric and behavioral comorbidities, particularly substance use disorders, are relatively common in patients with psoriasis. These findings support the need for regular mental health screening and integrated care approaches in psoriasis management.

## 1. Introduction

Psoriasis is a chronic inflammatory immune-mediated disorder, clinically characterized by erythematous, well-demarcated scaly plaques predominantly distributed on the elbows, knees, and scalp [1]. Although global psoriasis prevalence is estimated at approximately 2–3% [2], a Romanian study of 1500 patients reported a notably higher prevalence of 4.99%, highlighting significant regional variations [3]. Psoriasis is increasingly understood as a systemic condition with a diverse comorbidity profile that extends beyond the skin. These include metabolic, rheumatologic, gastrointestinal, infectious, and malignant diseases, as well as a growing number of psychiatric and behavioral disorders [4]. Psychodermatology is an emerging interdisciplinary field that explores the complex interactions between the skin and the mind, focusing both on how psychological factors can influence the onset or course of dermatological conditions, and on how these conditions, in turn, can affect patients’ mental health. As the field continues to evolve, dedicated psychodermatology clinics have begun to emerge, providing integrated care through multidisciplinary teams composed of dermatologists, psychiatrists, and psychologists [5,6]. The consistent presence of mental health issues in psoriasis is associated with reduced quality of life, more severe disease, and poor treatment adherence, which can compromise clinical outcomes and increase healthcare costs due to prolonged disease activity and frequent treatment adjustments [7]. The prevalence of psychiatric comorbidities in patients with psoriasis differs widely across countries, likely due to a combination of factors such as environmental stress, socioeconomic conditions, cultural views on mental health, and differences in access to specialized care. According to data from the Global Healthcare Study on Psoriasis (GHSP), depression rates among patients with psoriasis were 20.3% in the United States, 18.5% in Brazil, 16.1% in Chile, 7.4% in Switzerland, 1.7% in Singapore, and 0% in China, highlighting the wide range in the prevalence of mental health disorders [8].

Stress is widely recognized as a key trigger of the immune dysregulation underlying psoriasis, contributing to both disease onset and chronicity. Conversely, psoriasis imposes a significant psychological burden, characterized by elevated stress, depression, anxiety, low self-esteem, and an increased risk of addictive behaviors, often driven by social stigma and isolation. These bidirectional interactions create a self-perpetuating cycle that exacerbates both the immunological and psychosocial burden of the disease [9].

The pathogenesis of psoriasis involves a complex interplay of immune cells and cytokines, notably the interactions among IL-23-producing dendritic cells, IL-17-secreting Th17 lymphocytes, and activated keratinocytes. Central cytokines driving the chronic inflammation characteristic of psoriasis include IL-17, IL-22, TNF-α, and IL-6 [10].

Increasing attention has been given to psychiatric comorbidities, particularly depression and anxiety, which significantly elevate the overall burden of psoriasis. Patients with psoriasis are more likely to experience these psychiatric disorders compared to the general population [10,11]. Shared inflammatory mechanisms, particularly elevations in pro-inflammatory cytokines such as IL-6, TNF-α, and IL-17, underpin the connection between psoriasis and psychiatric disorders [10,12,13]. Indeed, psoriasis patients with depression exhibit higher systemic inflammation markers, including C-reactive protein and erythrocyte sedimentation rate, compared to non-depressed psoriasis patients [14]. Furthermore, comorbid depression amplifies systemic inflammation and increases cardiovascular risk, as evidenced by elevated vascular inflammation and coronary plaque burden [15].

Recent genetic analyses suggest a bidirectional relationship between psoriasis and depression, potentially mediated by IL-17A, a cytokine elevated in major depressive disorder [16,17]. Multiple studies have demonstrated a functional connection between the brain and the skin through the hypothalamic–pituitary–adrenal (HPA) axis, primarily mediated by corticotropin-releasing hormone (CRH). In response to stress, CRH stimulates the production of proinflammatory cytokines within the skin, subsequently activating keratinocytes and promoting the formation of psoriasis plaques. Additionally, increased CRH production has been associated with the development of depressive symptoms, further underscoring the bidirectional relationship between stress, inflammation, and psychiatric comorbidities in psoriasis [18]. Beyond biological mechanisms, the psychosocial impact of psoriasis, such as stigma, social isolation, and diminished self-esteem, significantly compromises patients’ quality of life, comparable to severe chronic illnesses [19,20,21].

Behavioral factors such as alcohol consumption further exacerbate psoriasis severity and negatively impact therapeutic responses. Excessive alcohol intake, prevalent in approximately one-third of psoriasis patients, is associated with increased disease severity, reduced treatment efficacy, and impaired quality of life [22], potentially through heightened cytokine production and disrupted keratinocyte function [23].

Similarly, smoking is a recognized risk factor influencing both psoriasis incidence and severity [24]. Epidemiological studies have consistently demonstrated higher risks of psoriasis in smokers, proportional to smoking intensity [25,26]. Smokers typically present with higher PASI scores, reflecting increased disease severity, and show diminished responses to biologic therapies [27,28]. Mechanistically, smoking exacerbates psoriasis through oxidative stress and activation of inflammatory pathways (NF-κB, JAK–STAT signaling), thus elevating key psoriasis-associated cytokines (TNF-α, IL-17, IL-23) [29,30]. Encouragingly, smoking cessation significantly reduces psoriasis risk and severity over time [31]. Accordingly, we conducted a cross-sectional study at a tertiary dermatology center in Iași, Romania, to quantify the prevalence of major psychiatric and behavioral comorbidities, particularly substance addictions, among patients with psoriasis and evaluate their demographic and clinical correlates, including disease severity. By elucidating these associations, we aim to raise awareness, promote holistic management strategies, and reduce the stigma surrounding mental health screening in dermatology.

## 2. Materials and Methods

### 2.1. Study Design and Participants

We conducted a cross-sectional observational study based on retrospective data collected from 316 patients with histopathologically confirmed psoriasis who were admitted to the Dermatology Clinic of the Clinical Railway Hospital in Iași, Romania, between January 2021 and March 2025. The study included both continuous inpatients and day-care admissions. No age restrictions were applied, and both adult and pediatric patients were eligible. Patients were selected through medical record review, based on the availability of complete clinical and psychiatric data relevant to the study. Coexisting dermatologic conditions were not exclusion criteria. Exclusion criteria included lack of histopathologic confirmation of psoriasis and incomplete demographic, clinical, or psychiatric documentation.

All figures and tables in this study are based exclusively on the subset of psoriasis patients identified with psychiatric and/or behavioral comorbidities.

### 2.2. Data Collection

We retrospectively analyzed medical records from patients evaluated over a four-year period, between January 2021 and March 2025. Demographic, clinical, and behavioral data were collected from patients’ medical records and structured clinical evaluation forms. Psoriasis severity was assessed using the Psoriasis Area and Severity Index (PASI). Patients were categorized as having mild-to-moderate psoriasis if PASI < 10, and moderate-to-severe psoriasis if PASI ≥ 10, in accordance with international clinical practice guidelines. Disease duration was calculated based on the self-reported onset of initial psoriatic lesions, rather than from the date of histopathological confirmation.

Psychiatric diagnoses such as depression and anxiety were identified based on pre-existing documentation in patient medical records. In contrast, addictive behaviors (including alcohol, tobacco use, and binge eating) were assessed using validated screening tools, given that formal psychiatric diagnoses for these conditions were rarely available.

Initial clinical anamnesis revealed no illicit drug use, and no eating disorders were identified beyond binge eating. Consequently, no patients with other eating disorders, such as bulimia nervosa or anorexia nervosa, were included in the cohort.

Alcohol use was assessed using the Alcohol Use Disorders Identification Test-Concise (AUDIT-C). Only individuals who met the threshold for hazardous alcohol consumption (AUDIT-C ≥4 for men and ≥3 for women) were included, while occasional or recreational use was excluded.

Tobacco dependence was evaluated with the Fagerström Test for Nicotine Dependence, and only patients demonstrating moderate-to-high dependence (Fagerström Test scores ≥ 5)—aligned with DSM-5 criteria for tobacco use disorder—were included. Social or occasional smoking did not meet the inclusion criteria.

Binge eating behavior was assessed using the Binge Eating Scale (BES), with patients scoring ≥18 (moderate-to-severe range) classified as having compulsive eating behavior. This classification was based solely on BES scores and did not involve a formal psychiatric diagnosis.

These instruments are validated tools commonly used to screen for alcohol use, nicotine dependence, and binge eating across diverse populations. Although they are not specifically validated for patients with psoriasis, their widespread use in clinical and dermatological settings supports their applicability for assessing behavioral comorbidities in this context. In all these cases, patients were advised to seek specialized psychological or psychiatric support.

### 2.3. Statistical Analysis

Statistical analyses were conducted using IBM SPSS Statistics, version 18.0 (IBM Corp., Armonk, NY, USA). The objective was to evaluate associations between psoriasis severity and various socio-demographic, behavioral, and psychiatric factors.

Descriptive statistics were applied to summarize all variables. The normality of continuous variables was assessed using the Skewness test, with values between −2 and +2 considered acceptable. Parametric comparisons between groups were performed using Student’s *t*-test for two groups and analysis of variance (ANOVA) for more than two groups. Categorical variables were compared using the Chi-square (χ^2^) test.

Pearson’s correlation coefficient (r) was calculated to assess linear relationships between continuous variables. A binary logistic regression model using the Forward Likelihood Ratio (LR) method was applied to identify independent predictors of moderate-to-severe psoriasis. Results were reported as Odds Ratios (OR) with corresponding 95% Confidence Intervals (CI). A two-tailed *p*-value < 0.05 was considered statistically significant. Patients with missing data on key demographic, clinical, or psychiatric variables were excluded from the final analysis.

## 3. Results

### 3.1. Cohort Overview

The study included 316 patients diagnosed with psoriasis, with a mean age of 49.5 years (range: 8 to 84), of whom 52.8% were male and 47.2% female. Regarding disease severity, 204 patients (64.6%) had moderate-to-severe psoriasis (PASI ≥ 10), while 112 (35.4%) had mild-to-moderate forms (PASI < 10). Most patients (61.4%) resided in urban areas, while 38.6% were from rural settings.

### 3.2. Subgroup of Patients with Psychiatric and Behavioral Comorbidities

Out of the total sample, 88 patients (27.8%) were identified as having at least one psychiatric or behavioral comorbidity. This cohort had a mean age of 52 ± 17 years and was predominantly male (67.0%). Most patients (88.6%) in this group had moderate-to-severe psoriasis.

**Figure 1:**
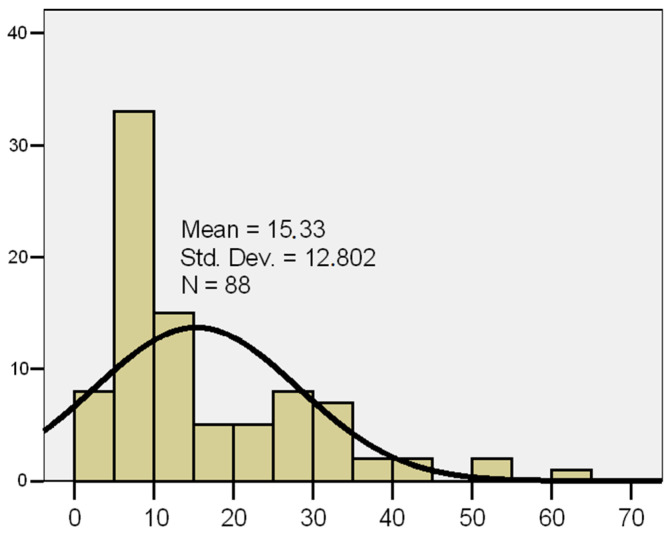
Histogram of disease duration in patients with psoriasis. Note: *X*-axis: disease duration (years); *Y*-axis: number of patients (*N*).

Disease duration ranged from 2 to 63 years, with a mean of 15.3 ± 12.8 years and a median of 10 years. The distribution showed a slight positive skew (skewness = 1.43), which allowed the use of parametric statistical tests. The distribution of disease duration is illustrated in Figure 1.

Age values ranged from 9 to 79 years, with a mean of 51.83 ± 17.35 years and a median of 55 years. The distribution was homogeneous and suitable for parametric testing, as confirmed by the skewness test (skewness = –0.823; as can be seen in Table 1).

**Figure A1:**
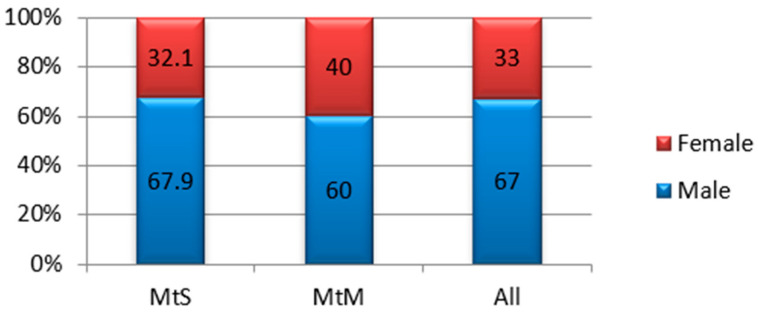
Sex distribution by psoriasis severity (MtS vs. MtM). MtS = moderate-to-severe psoriasis (PASI ≥ 10); MtM = mild-to-moderate psoriasis (PASI < 10). The figure shows the proportion of male and female patients in each severity group.

The patient population was predominantly male (67%), with a male-to-female ratio of 2:1. There was no significant difference in sex distribution according to psoriasis severity (67.9% in the moderate-to-severe group vs. 60.0% in the mild-to-moderate group; *p* = 0.430) (see Appendix A, Figure A1; Table 2).

**Figure A2:**
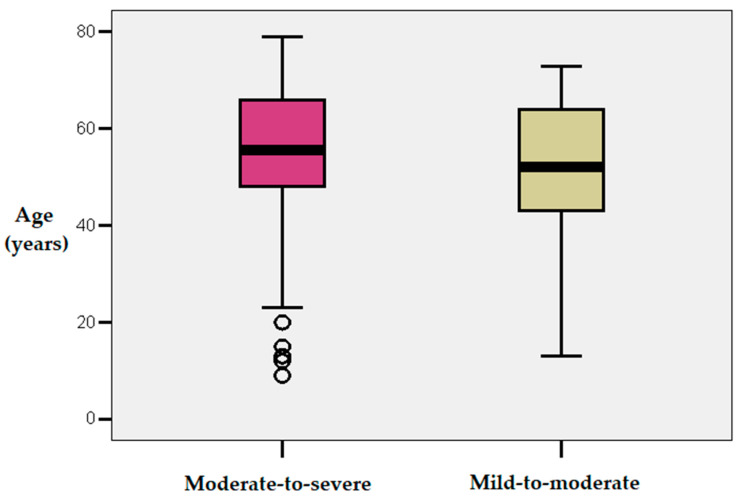
Mean age by psoriasis severity.

According to disease severity, patients with moderate-to-severe psoriasis had a slightly higher mean age compared to those with mild-to-moderate disease (52.10 vs. 49.70 years; *p* = 0.683) (see Appendix A, Figure A2).

**Figure A3:**
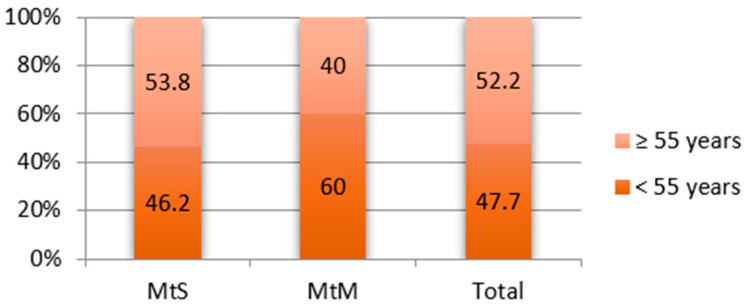
Age group distribution by psoriasis severity (MtS vs. MtM). MtS = moderate-to-severe psoriasis; MtM = mild-to-moderate psoriasis. No statistically significant difference was observed (53.8% in MtS vs. 40.0% in MtM; *p* = 0.312).

Patients aged over 55 years accounted for 52.3% of the entire cohort. No significant difference was observed between the moderate-to-severe and mild-to-moderate psoriasis groups (53.8% vs. 40.0%; *p* = 0.312) (see Appendix A, Figure A3; Table 2).

**Figure 2:**
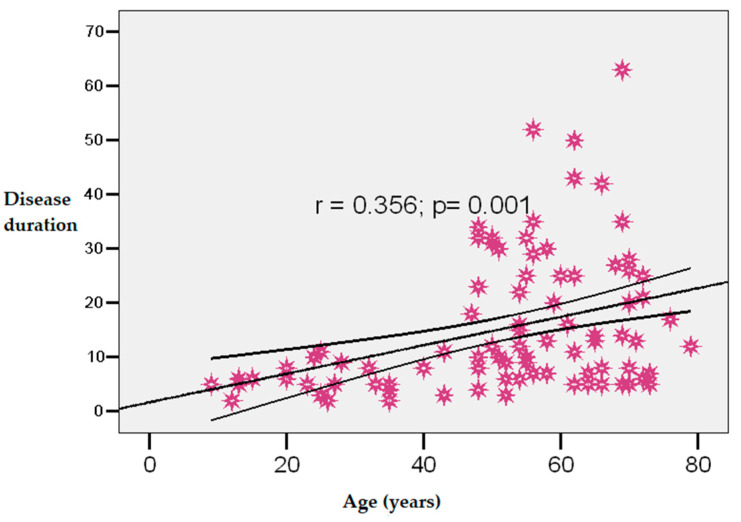
Correlation between age and psoriasis duration.

A moderate positive correlation was observed between age and disease duration (r = 0.356; *p* = 0.001), as can be seen in Figure 2.

**Figure A4:**
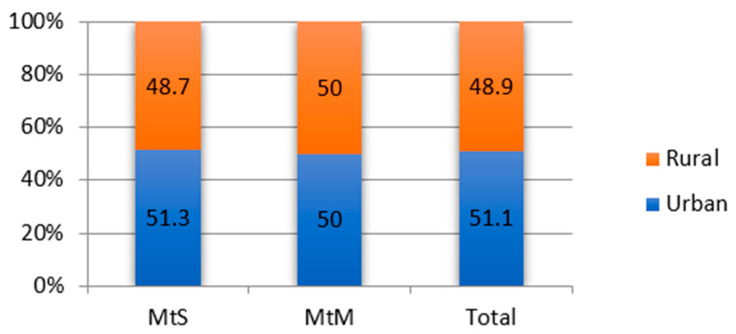
Distribution of urban and rural residence by psoriasis severity. No statistically significant difference was observed. MtS = moderate-to-severe psoriasis; MtM = mild-to-moderate psoriasis.

Within the study sample, the distribution by area of residence did not differ significantly according to psoriasis severity (51.3% in the moderate-to-severe group vs. 50.0% in the mild-to-moderate group; *p* = 0.601) (see Appendix A, Figure A4; Table 2).

### 3.3. Psychiatric and Behavioral Comorbidities

**Figure 3:**
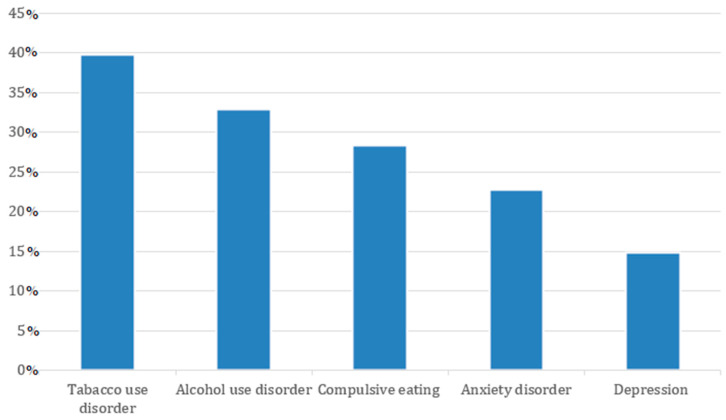
Prevalence of psychiatric and behavioral comorbidities.

The most commonly reported psychiatric and behavioral conditions included tobacco use disorder (39.8%), alcohol use disorder (32.9%), compulsive eating behavior (28.4%), anxiety disorders (22.7%), and depressive disorders (14.8%) (Figure 3). When considering the entire psoriasis cohort (*n* = 316), these conditions correspond to prevalence rates of 11.1%, 9.2%, 7.9%, 6.3%, and 4.1%, respectively. Additional diagnoses included personality disorders, dementia, post-traumatic stress disorder (PTSD), and sleep disorders.

**Figure A5:**
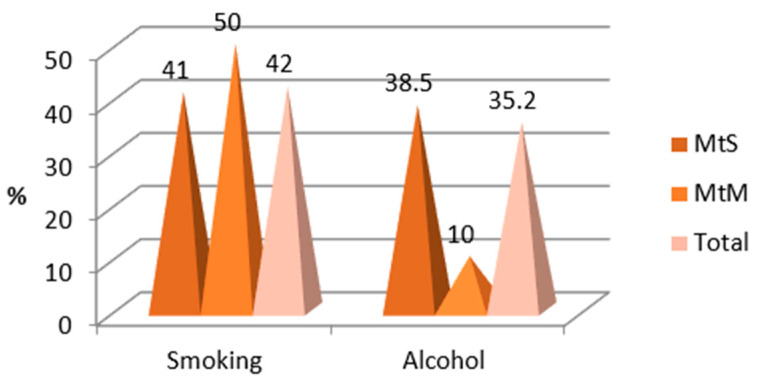
Smoking and alcohol misuse by psoriasis severity (MtS vs. MtM)**.** MtS = moderate-to-severe psoriasis; MtM = mild-to-moderate psoriasis.

In the moderate-to-severe group, 41.0% were smokers and 38.5% reported alcohol use, compared to 50.0% and 10.0%, respectively, in the mild-to-moderate group (*p* = 0.415 for smoking; *p* = 0.050 for alcohol) (see Appendix A, Figure A5; Table 2).

**Figure A6:**
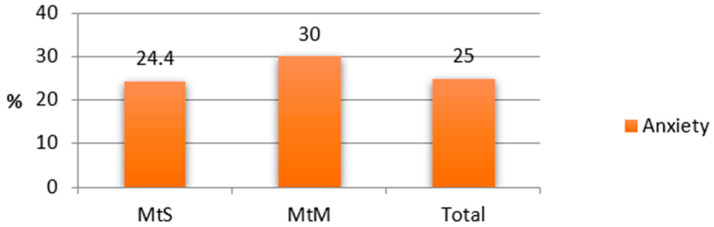
Anxiety by psoriasis severity. MtS = moderate-to-severe psoriasis; MtM = mild-to-moderate psoriasis.

Anxiety (30.0% vs. 24.4%; *p* = 0.479) and compulsive eating behavior (30.0% vs. 28.2%; *p* = 0.583) were slightly more frequent among patients with mild-to-moderate psoriasis (see Appendix A, Figure A6; Table 2).

**Figure A7:**
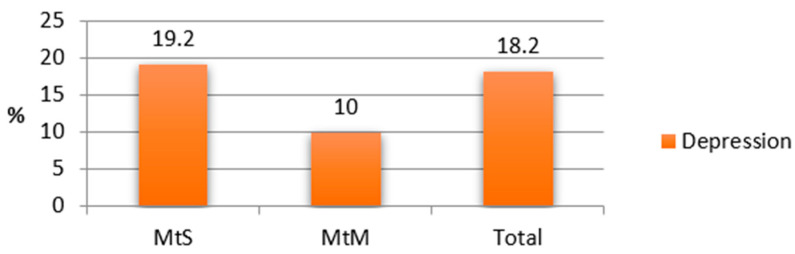
Depression by psoriasis severity. MtS = moderate-to-severe psoriasis; MtM = mild-to-moderate psoriasis.

**Figure A8:**
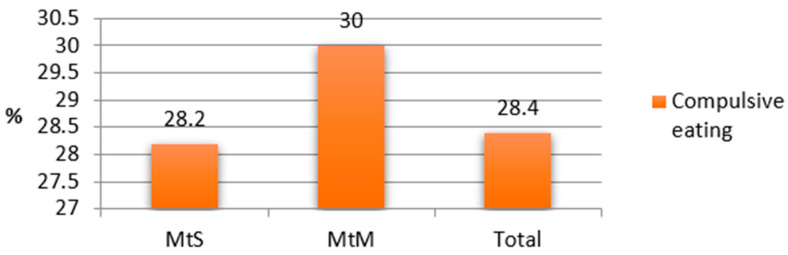
Compulsive eating by psoriasis severity. MtS = moderate-to-severe psoriasis; MtM = mild-to-moderate psoriasis.

In contrast, depressive symptoms (19.2% vs. 10.0%; *p* = 0.421) and other psychiatric disorders (15.4% vs. 10.0%; *p* = 0.545) were more prevalent in the moderate-to-severe group (see Appendix A, Figure A7 and Figure A8; Table 2).

These findings are further detailed in Table 2, which presents their distribution relative to psoriasis severity.

**Figure A9:**
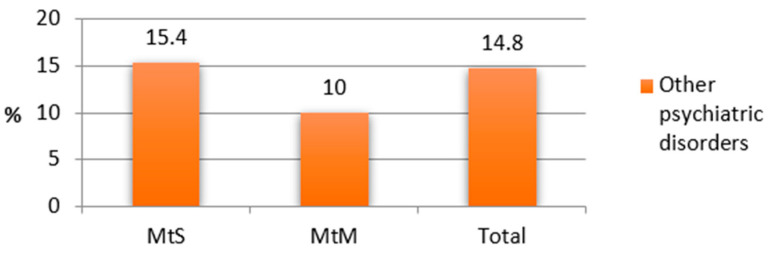
Other psychiatric disorders by psoriasis severity. MtS = moderate-to-severe psoriasis; MtM = mild-to-moderate psoriasis.

Less frequently observed psychiatric diagnoses included dementia (*n* = 3), sleep disturbances (*n* = 5), post-traumatic stress disorder (*n* = 2), personality disorders (*n* = 2), suicidal ideation (*n* = 1), and one case involving epilepsy, cognitive impairment, and cerebral leukoaraiosis. These conditions were grouped under the category of “other psychiatric disorders”, whose distribution by psoriasis severity is illustrated in Figure A9.

A multivariate logistic regression analysis was conducted to evaluate the probability of developing moderate-to-severe psoriasis in relation to several potential risk factors, including sex, age, area of residence, addictive behaviors, and psychiatric history.

Increased odds for moderate-to-severe psoriasis were observed in male patients (OR = 1.318; 95% CI: 0.332–5.233; *p* = 0.695), those living in urban areas (OR = 1.826; 95% CI: 0.472–7.064; *p* = 0.383), smokers (OR = 1.620; 95% CI: 0.320–8.199; *p* = 0.560), individuals with anxiety (OR = 1.854; 95% CI: 0.369–9.329; *p* = 0.454), depression (OR = 2.257; 95% CI: 0.217–23.450; *p* = 0.495), compulsive eating behavior (OR = 2.827; 95% CI: 0.586–13.634; *p* = 0.195), and other psychiatric disorders (OR = 4.019; 95% CI: 0.442–36.537; *p* = 0.217).

A statistically significant association was observed between alcohol use disorder and psoriasis severity (*p* = 0.037), with an odds ratio of 10.971 (95% CI: 1.144–15.208). Detailed results are presented in Table 3.

## 4. Discussion

The findings of this study reinforce the growing recognition that psoriasis is not solely a dermatologic condition, but rather a systemic inflammatory disease with significant psychiatric and behavioral dimensions, as reflected in its strong associations with depression, anxiety, and impaired psychological well-being [32]. To support a clearer interpretation of our findings, we differentiate between psychiatric disorders, such as mood and anxiety conditions, and behavioral patterns like substance use and compulsive eating. These groups are often linked but represent different clinical challenges.

In our cohort, 27.8% of patients presented with at least one psychiatric or behavioral comorbidity. The most frequently observed comorbidities were tobacco use disorder (11.1%), alcohol use disorder (9.2%), binge eating behavior (7.9%), anxiety disorders (6.3%), and depression (4.1%). While these rates clearly illustrate the burden of psychosocial and behavioral dysfunction among psoriasis patients, they are consistently lower than those reported in prior studies. These differences merit closer analysis, both in terms of methodological considerations and population-specific characteristics.

When focusing on depression, our data revealed a predominance of male patients (76.9%), with a lower mean age compared to the overall cohort. This finding diverges from results reported by Duvetorp et al. in a large-scale Swedish population study, in which female sex and younger age were associated with higher risk of depression in psoriatic patients [33]. These discrepancies may reflect differences in population size, cultural stigma surrounding mental health, or underdiagnosis in certain subgroups.

Fleming et al. conducted a systematic review of observational studies and found that anxiety prevalence among psoriasis patients ranged from 7% to 48%, depending on the population and instruments used [34]. Our lower rate of 6.3% likely reflects the fact that we included only clinically diagnosed anxiety disorders, as opposed to subclinical symptoms captured by psychometric scales. As a result, it is possible that anxiety is underreported in our sample due to lack of structured screening at the dermatological level.

Alcohol use disorder was the only factor significantly associated with psoriasis. Furthermore, it was linked to higher PASI scores in our cohort, in line with previous research [35]. Alcohol use disorder was reported in 14.9% of patients with psoriasis in a large German multicenter study [36], and as high as 30.6% in a UK cohort screened with the AUDIT questionnaire [37]. In contrast, our rate of 9.2% was notably lower. One likely explanation lies in our strict diagnostic criteria. Additionally, this lower prevalence may partially reflect social desirability bias, as participants may have underreported alcohol-related problems due to stigma or embarrassment. Furthermore, disparities in access to healthcare services may also contribute to the variability observed between studies.

In our study, the prevalence of tobacco misuse was 11.1% of the total cohort, notably lower than the 38.8% smoking rate reported in a 2024 real-world study from China [27]. This discrepancy reflects a difference in scope: the Chinese study considered all forms of smoking, including occasional and regular use, whereas our analysis focused specifically on individuals meeting diagnostic criteria for tobacco use disorder. This targeted approach was chosen to identify clinically relevant patterns of substance use, rather than general smoking behavior. Interestingly, within our cohort, tobacco misuse was more prevalent among patients with mild to moderate psoriasis compared to those with more severe forms. One possible explanation is that patients with advanced disease may shift toward other forms of dependence, such as alcohol, or may have received more consistent medical supervision and lifestyle counseling, including consistent advice to quit smoking. However, given the limited sample size, these interpretations remain speculative, and no definitive conclusions can be drawn at this stage.

Binge eating behavior, often underexplored in psoriatic populations, was present in 7.9% of our patients—a figure that closely aligns with the 7% reported by Altunay et al. in a Turkish study investigating the association between eating disorders and metabolic syndrome in psoriasis patients using the Eating Attitudes Test. This convergence is particularly interesting, given the relative scarcity of literature on eating disorders in psoriasis. Emerging evidence suggests that disordered eating, through its association with psychiatric comorbidities, may contribute to increased cardiovascular risk, warranting further investigation [38].

Taken together, our findings suggest that behavioral patterns, such as substance use and compulsive eating, deserve greater attention in clinical practice, alongside depression and anxiety. These behaviors may not only affect treatment adherence and overall quality of life, but also influence systemic inflammation and disease course. Moreover, their presence may reflect broader psychosocial burdens, such as stigma, social withdrawal, and maladaptive coping mechanisms [39].

The relatively lower prevalences observed in our study could be partially explained by the fact that many patients, especially those with moderate-to-severe forms of psoriasis, were undergoing systemic treatment, such as biologic agents, that maintained their PASI scores at minimal levels. This likely reflects a low inflammatory status at the time of evaluation, which may have attenuated the psychological burden. This observation aligns with numerous studies that have demonstrated a positive correlation between the severity of psoriasis and the intensity of depressive and anxiety symptoms among patients [40]. On the other hand, the absence of structured psychiatric screening and reliance on existing clinical diagnoses may have led to the under-recognition of subclinical symptoms, especially in the domains of anxiety and mood disturbance.

This study is subject to several limitations that should be taken into consideration. Firstly, given the cross-sectional nature of this study, we were unable to determine whether the psychiatric or behavioral conditions occurred before or after the onset of psoriasis; thus, no temporal or causal relationships can be confirmed. Secondly, we relied on existing diagnoses from patient files and interviews for depression and anxiety, without using specific psychological tests. This may have contributed to an underestimation of subclinical or undiagnosed psychiatric symptoms in these areas. Additionally, the study was conducted in a single tertiary dermatology center, which may limit the generalizability of the findings to broader populations. It is also important to acknowledge the possible influence of social desirability bias, especially in self-reported behaviors such as substance use. In addition, limited patient education on mental health may have made it more difficult for individuals to recognize or communicate their symptoms, which could have led to underdiagnosis.

Considering the key role of IL-17 and IL-23 cytokines in both the immunopathogenesis of psoriasis and the development of psychiatric symptoms such as depression and anxiety, future studies should investigate the potential benefits of biologic therapies targeting these pathways. Agents that inhibit IL-17 or IL-23 may not only improve dermatological outcomes, but also positively impact psychiatric comorbidities through systemic anti-inflammatory effects [41]. To substantiate these hypotheses, prospective longitudinal studies are needed to follow cohorts of psoriasis patients with psychiatric comorbidities both before and after treatment initiation, in order to assess the evolution of these conditions over time. Moreover, larger cross-sectional studies with more diverse and clinically complex patient populations are needed to better understand these associations and to identify subgroups who may benefit most from integrated, targeted treatment approaches.

Considering the relevance of these findings, dermatologic care should incorporate systematic mental health screening, as recommended by international guidelines such as those from the American Academy of Dermatology (AAD) and the European Academy of Dermatology and Venereology (EADV) [42,43].

## 5. Conclusions

Our findings show that psychiatric and behavioral comorbidities are common in patients with psoriasis, emphasizing the need for multidisciplinary care. Even though the rates of anxiety, substance use disorders, and binge eating were lower than in other studies, this may be due to differences in patient characteristics or diagnostic criteria. Maladaptive behaviors like alcohol misuse and tobacco dependence may affect treatment adherence and disease control. Using screening tools like AUDIT-C in routine dermatology visits could help identify these issues earlier. Finally, since IL-17 and IL-23 are involved in both psoriasis and some psychiatric conditions, future research should explore whether biologic therapies targeting these pathways can also improve mental health.

Given the substantial prevalence of psychiatric and behavioral comorbidities among patients with psoriasis, incorporating routine mental health screening into dermatologic practice is essential. This integrative approach can greatly enhance therapeutic strategies, support treatment adherence, and ultimately improve patient outcomes and quality of life.

## Figures and Tables

**Table 1:** Descriptive statistics for patient age (*N* = 88).

*N*	88
Mean	51.83
Median	55
Standard deviation	17.35
Variance	33.47
Skewness	−0.823
Standard Error Skewness	0.257
Minimum	9
Maximum	79
25th percentile	44
50th percentile	55
75th percentile	66

**Table 2:** Distribution of socio-demographic characteristics and psychiatric or behavioral disorders by psoriasis severity.

Characteristics	*N* (%)	Psoriasis Severity *				Chi^2^ Test *p*	RR	95% IC
		Moderate-to-Severe (*n* = 78)		Mild-to-Moderate (*n* = 10)				
		*n*	%	*n*	%			
Sex								
Male	59 (67.0%)	53	67.9	6	60.0	0.430	1.04	0.88–1.23
Female	29 (33.0%)	25	32.1	4	40.0			
Age Group								
<55 years	42 (47.7%)	36	46.2	6	60.0	0.312	0.94	0.81–1.09
≥55 years	46 (52.3%)	42	53.8	4	40.0			
Residence								
Urban	45 (51.1%)	40	51.3	5	50.0	0.601	1.01	0.87–1.17
Rural	43 (48.9%)	38	48.7	5	50.0			
Psychiatric or behavioral disorders								
Tobacco use disorder	37 (42.0%)	32	41.0	5	50.0	0.415	0.96	0.82–1.12
Alcohol use disorder	31 (35.2%)	30	38.5	1	10.0	0.050	1.15	1.01–1.31
Anxiety	22 (25.0%)	19	24.4	3	30.0	0.479	0.97	0.80–1.16
Depression	16 (18.2%)	15	19.2	1	10.0	0.421	1.07	0.92–1.25
Compulsive eating	25 (28.4%)	22	28.2	3	30.0	0.583	0.99	0.84–1.17
Other	13 (14.8%)	12	15.4	1	10.0	0.545	1.05	0.88–4.64

* Psoriasis severity was categorized using PASI scores: mild-to-moderate psoriasis was defined as PASI < 10; moderate-to-severe as PASI ≥ 10. Abbreviations: RR—Risk Ratio; 95% CI—95% Confidence Interval; *p*—*p*-value; Chi^2^ Test—Chi-squared Test.

**Table 3:** Binary logistic regression model for predicting moderate-to-severe psoriasis based on socio-demographic and psychiatric variables.

Independent Variable	B	S.E.	*p*-Value	Odds Ratio (Exp B)	CI95	
					95% CI Lower	95% CI Upper
Male sex	0.276	0.70	0.695	1.318	0.332	5.233
Age < 55 years	0.027	0.686	0.968	1.028	0.268	3.946
Urban residence	0.602	0.690	0.383	1.826	0.472	7.064
Smoking use disorder	0.483	0.827	0.560	1.620	0.320	8.199
Alcohol use disorder	2.395	1.153	0.037 *	10.971	1.144	15.208
Anxiety disorder	0.618	0.824	0.454	1.854	0.369	9.329
Depression	0.814	1.194	0.495	2.257	0.217	23.450
Compulsive eating	1.039	0.803	0.195	2.827	0.586	13.634
Other psychiatric disorders	1.391	1.126	0.217	4.019	0.442	36.537

Note: * Bold values indicate statistically significant results (*p* < 0.05).

## Data Availability

The original contributions presented in this study are included in the article. Further inquiries can be directed at the corresponding authors.

## Abbreviations

The following abbreviations are used in this manuscript:

AAD	American Academy of Dermatology
ANOVA	Analysis of Variance
AUDIT-C	Alcohol Use Disorders Identification Test—Concise
BES	Binge Eating Scale
CI	Confidence Interval
CRH	Corticotropin-Releasing Hormone
DSM-5	Diagnostic and Statistical Manual of Mental Disorders, Fifth Edition
EADV	European Academy of Dermatology and Venereology
HPA	Hypothalamic–Pituitary–Adrenal axis
IL	Interleukin
JAK–STAT	Janus kinase–signal transducer and activator of transcription pathway
NF-κB	Nuclear Factor kappa-light-chain-enhancer of activated B cells
OR	Odds Ratio
PASI	Psoriasis Area and Severity Index
PTSD	Post-Traumatic Stress Disorder
RR	Relative Risk
SD	Standard Deviation
SPSS	Statistical Package for the Social Sciences
TNF-α	Tumor Necrosis Factor-alpha
